# Timely Estimates of 5-Year Relative Survival for Patients With Cervical Cancer: A Period Analysis Using Cancer Registry Data From Taizhou, Eastern China

**DOI:** 10.3389/fpubh.2022.926058

**Published:** 2022-07-25

**Authors:** Hongsheng Lu, Lu Li, Yongran Cheng, Zhaohui Yang, Xuequan Cao, Hui Zhang, Dongju Qiao, Liangyou Wang, Tianhui Chen

**Affiliations:** ^1^Department of Pathology, Taizhou Central Hospital (Taizhou University Hospital), Taizhou, China; ^2^College of Pharmaceutical Sciences, Zhejiang University, Hangzhou, China; ^3^School of Public Health, Hangzhou Medical College, Hangzhou, China; ^4^Department of Non-communicable Chronic Disease Control and Prevention, Taizhou Municipal Center for Disease Control and Prevention, Taizhou, China; ^5^Department of Cancer Prevention/Zhejiang Cancer Institute, Cancer Hospital of the University of Chinese Academy of Sciences (Zhejiang Cancer Hospital), Hangzhou, China; ^6^Institute of Basic Medicine and Cancer (IBMC), Chinese Academy of Sciences, Hangzhou, China; ^7^Department of Preventative Medicine, School of Medicine, Ningbo University, Ningbo, China

**Keywords:** cervical cancer, cancer registry, survival, 5-year relative survival, period analysis

## Abstract

**Objectives:**

While timely assessment of long-term survival for patients with cervical cancer is essential for the evaluation of early detection and screening programs for cervical cancer, those data are extremely scarce in China. We aimed to timely and accurately assess long-term survival for patients with cervical cancer in eastern China, using cancer registry data from Taizhou, eastern China.

**Methods:**

Patients diagnosed with cervical cancer during 2004–2018 from four cancer registries with high-quality data from Taizhou, eastern China were included. A period analysis was used to calculate the 5-year relative survival (RS) overall and on stratification by sex, age at diagnosis, and region. Additionally, the projected 5-year relative survival (RS) of patients with cervical cancer during 2019–2023 was evaluated, using a model-based period analysis.

**Results:**

Overall 5-year RS for patients with cervical cancer during 2014–2018 reached 90.9%. When stratified by age at diagnosis, we found a clear age gradient for 5-year RS, declining from 95.6% for age <45 years to 68.7% for age >74 years, while urban areas had higher 5-year RS compared to rural areas (92.9 vs. 88.6%). We found a clear increasing trend of 5-year RS during 2004–2018 overall and on stratification by region and age at diagnosis. The projected overall 5-year RS is expected to reach 94.2% for the period 2019–2023.

**Conclusions:**

We found that, for the first time in China, using period analysis, the most up-to-date (during 2014–2018) 5-year RS for patients with cervical cancer reached 90.9%. Our data have important implications for the timely evaluation of early detection and screening programs for patients with cervical cancer in eastern China.

## Introduction

Cervical cancer ranks as the second most common type of cancer in women and predominantly occurs in postmenopausal women in less developed countries (1–3). According to estimates by GLOBOCAN 2018, ~570,000 women developed cervical cancer and an estimated 311,000 deaths occurred in 2018 (3.2% of all female cancer cases) (4). Cervical cancer is also one of the most common types of cancer in women in China, comprising one-third of global new cases of cervical cancer (5). While the incidence of cervical cancer in China increased from 3.06 per 100,000 in 1989 to 15.3 per 100,000 in 2014 (6, 7), a high incidence rate of cervical cancer was found in rural areas of the central and western regions of China (2, 7, 8), which could be mainly attributed to the absence of effective screening and vaccinations against human papillomavirus (HPV) before 2017.

Cervical cancer is associated with HPV infection due to sexual activity and reproductive factors (1, 2, 9). Declining incidence and mortality rates of cervical cancer are mainly found in Europe, Australia/New Zealand, and North America (4), which could be mainly due to the successful implementation of population-based cytological screening programs. Long-term survival estimates assessed using population-based cancer registry data are essential for the evaluation of cancer burden. A 5-year relative survival (RS) is the most important index for assessing cancer burden and is essential for the evaluation of early detection and screening programs for cervical cancer, which shall be as up to date as possible. Studies of 5-year RS data on patients with cervical cancer mainly involved the Western population, such as Germany and the United Kingdom (1, 10, 11), while data on the Chinese population are scarce (12).

Because traditional cohort and complete methods use 5-year follow-up data, the approaches may have a 5-year delay, at least for survival estimates (in addition to other time requests for data collection, calculation, and publication). Period analysis, which does not require 5-year follow-up data to calculate survival estimates, is the gold standard for the assessment of long-term survival of patients with cancer using data from population-based cancer registries and has been widely used in the Western populations (10, 13–15). However, its application in China has been scarce. Our group found, for the first time, by systematically using period analysis and cancer registry data from eastern China, that period analysis is superior to traditional cohort and complete methods, which can provide more up-to-date precise estimates of long-term survival overall and on stratification by sex, age at diagnosis, region, and cancer sites (including cervical cancer) (16).

In the current study, we aimed to provide the most up-to-date (during 2014–2018) estimates of 5-year RS for patients with cervical cancer from the Chinese population using period analysis and population-based cancer registry data from Taizhou city, eastern China. We also aimed to predict 5-year RS for the upcoming 2019–2023 period using the data during 2004–2018 and a model-based period analysis.

## Methods

### Data Sources

**Figure 1:**
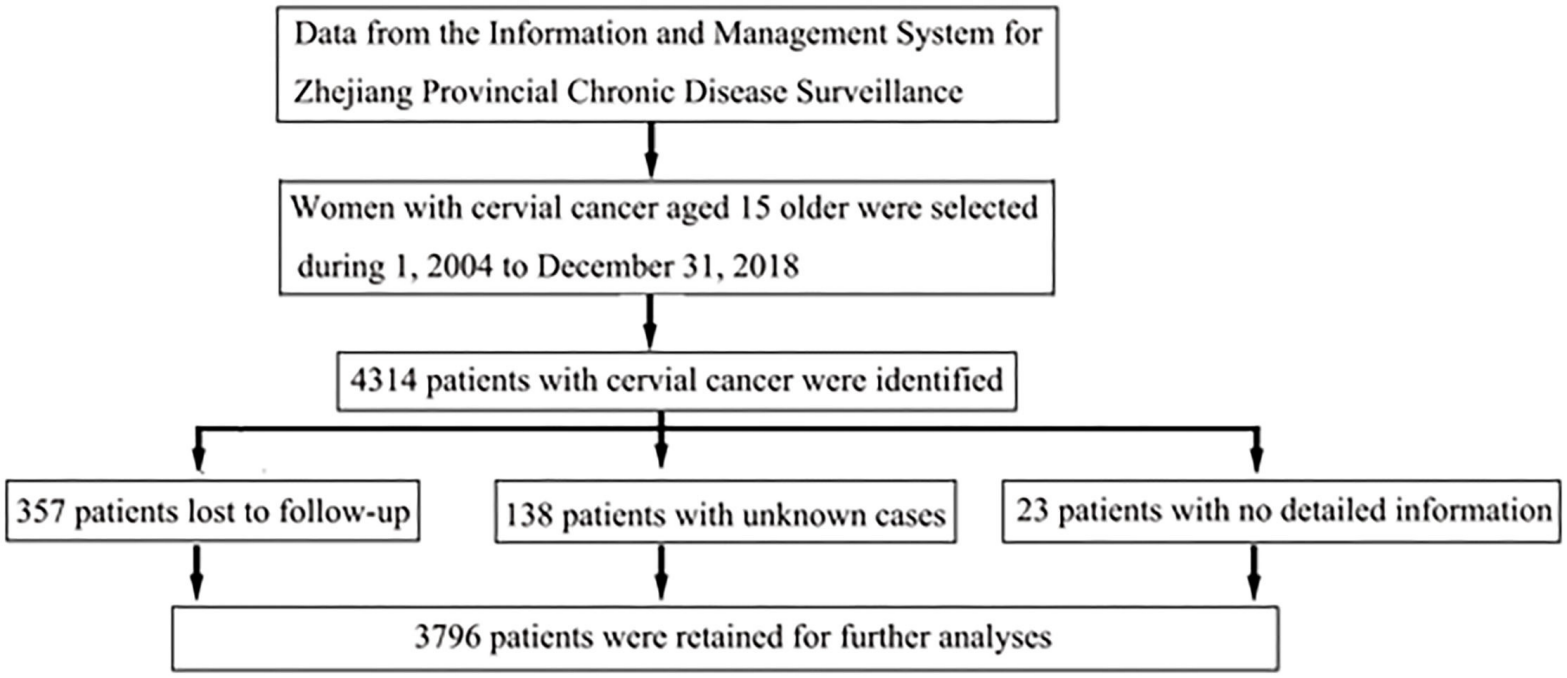
Flowchart of this study.

The Information and Management System for Zhejiang Provincial Chronic Disease Surveillance was a platform for monitoring the incidence and mortality rates of chronic diseases (including cancer) for inhabitants living in Zhejiang province. This population-based cancer surveillance system was used to assess incidence rates for cancer from nine registries in Taizhou. According to the inclusion criteria on the proportions of cases notified by death certificate only (DCO) under 13% throughout the study period, data from four (Luqiao, Yuhuan, Xianju, and Wenling) out of nine registries from the Taizhou region were included for further analyses. Women aged 15 or older when diagnosed with cervical cancer from 1 January 2004 to 31 December 2018 were included in this study. Mortality follow-up concerning vital status was available until the end of 31 December 2018. Using the ICD-10 code C53, we identified a total of 4,314 patients with cervical cancer, and among them, 357 patients lost to follow-up, 23 patients with no detailed information, and 138 patients with unknown cases were eventually excluded. Thus, 3,796 cases were retained for further analyses (Figure 1).

### Statistical Analyses

Throughout this study, relative survival (RS), which represents disease-specific survival within a patient population where cancer is the only cause of death, was used to present long-term survival estimates. The RS is derived as the ratio of absolute survival of patients with cancer to the expected survival of individuals corresponding to the patients with cancer with similar characteristics in the calendar period of observation. Throughout this article, 5-year RS estimates for patients with cervical cancer were calculated as the ratio of the observed survival in the patient group with cervical cancer and the expected survival from a comparable group in the general population (2) (Supplementary Material). Estimates of expected survival were derived using the Ederer II method from the life table of the population of four cities of Taizhou (Luqiao, Yuhuan, Xianju, and Wenling). The model-based period analysis makes full use of the data from the cancer registry system and improves the accuracy and timeliness of survival analysis. The analyses were carried out by use of the Generalized Linear Model (GLM) function and the package “period” of R version 3.13 (R Foundation for Statistical Computing, Vienna, Austria) (17), improving the accuracy and timeliness of period survival analysis.

## Results

### Basic Characteristics of Patients With Cervical Cancer

The basic characteristics of patients diagnosed with cervical cancer during 2004–2018 are presented in Table 1. Overall, we included 3,796 patients with cervical cancer, consisting of 287 cases during 2004–2008, 1,387 during 2009–2013, and 2,122 during 2014–2018. While the average age at diagnosis was 53.2 years, age at diagnosis <45 years accounted for only 25%, and 56% of the patients ranged from 45 to 64 years. Only 14% of patients were from an urban area, compared to 86% of them from a rural area.

**Table 1:** Basic characteristics of patients diagnosed with cervical cancer during 2004–2018 from Taizhou, eastern China.

**Characteristics**	**Number of cases (%)**	**Diagnosis period**		
		**2004–2008 (%)**	**2009–2013 (%)**	**2014–2018 (%)**
**Total**	3,796	287	1,387	2,122
**Region**
Urban area	537 (14%)	36 (13%)	287 (21%)	369 (17%)
Rural area	3,259 (86%)	251 (87%)	1,100 (79%)	1,753 (83%)
**Average age (years)**	53.2	51.8	52.3	53.8
**Age at diagnosis (years)**
<45	963 (25%)	76 (26%)	382 (28%)	505 (24%)
45–54	1,241 (33%)	69 (24%)	531 (38%)	641 (30%)
55–64	889 (23%)	91 (32%)	267 (19%)	531 (25%)
65–74	457 (12%)	36 (13%)	126 (9%)	295 (14%)
>74	246 (7%)	15 (5%)	81 (6%)	150 (7%)

### 5-Year Relative Survival for Patients With Cervical Cancer During 2014–2018

Overall, 5-year relative survival rate during 2014–2018 reached 90.9% (Table 2). When stratified by age at diagnosis, we found an age gradient, with 5-year RS decreasing to 95.6% for age <45 years and to 68.7% for age >74 years. Urban areas had a higher 5-year RS, compared to rural areas (92.9 vs. 88.6%).

**Table 2:** Five-year relative survival of patients with cervical cancer during 2014–2018 in Taizhou, eastern China.

	**5-Year relative survival (%)**	**Standard error**
**Overall**	90.9	1.2
**Age at diagnosis (years)**
<45	95.6	2.2
45–54	93.3	1.6
55–64	89.6	1.4
65–74	83.2	3.1
>74	68.7	3.3
**Region**
Urban area	92.9	1.5
Rural area	88.6	1.4

### Trends of 5-Year RS During 2004–2018 and Projection During 2019–2023

**Figure 2:**
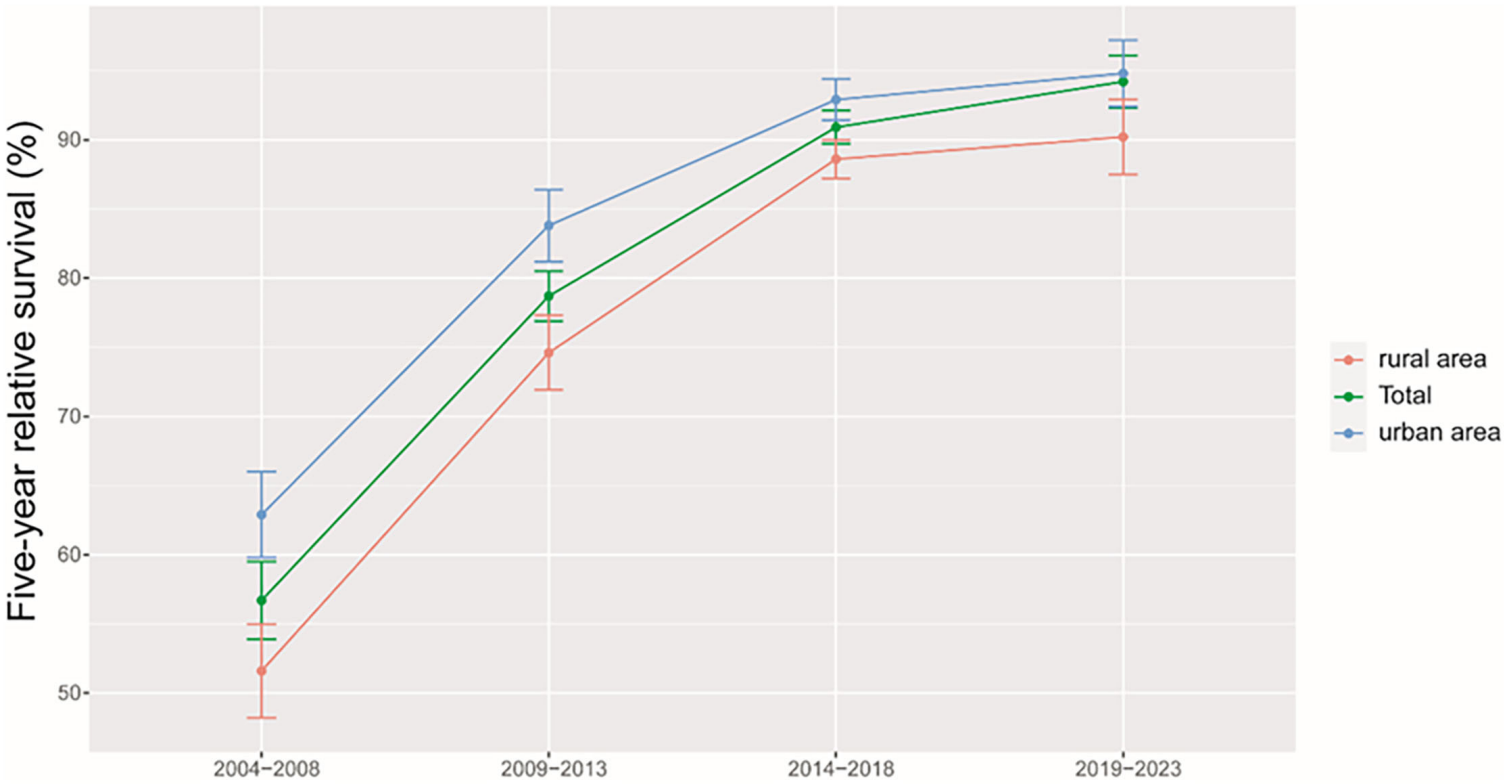
Trends of 5-year relative survival for patients with cervical cancer from Taizhou, eastern China during 2004–2008, 2009–2013, 2014–2018, and 2019–2023 by region (urban areas vs. rural areas).

**Figure 3:**
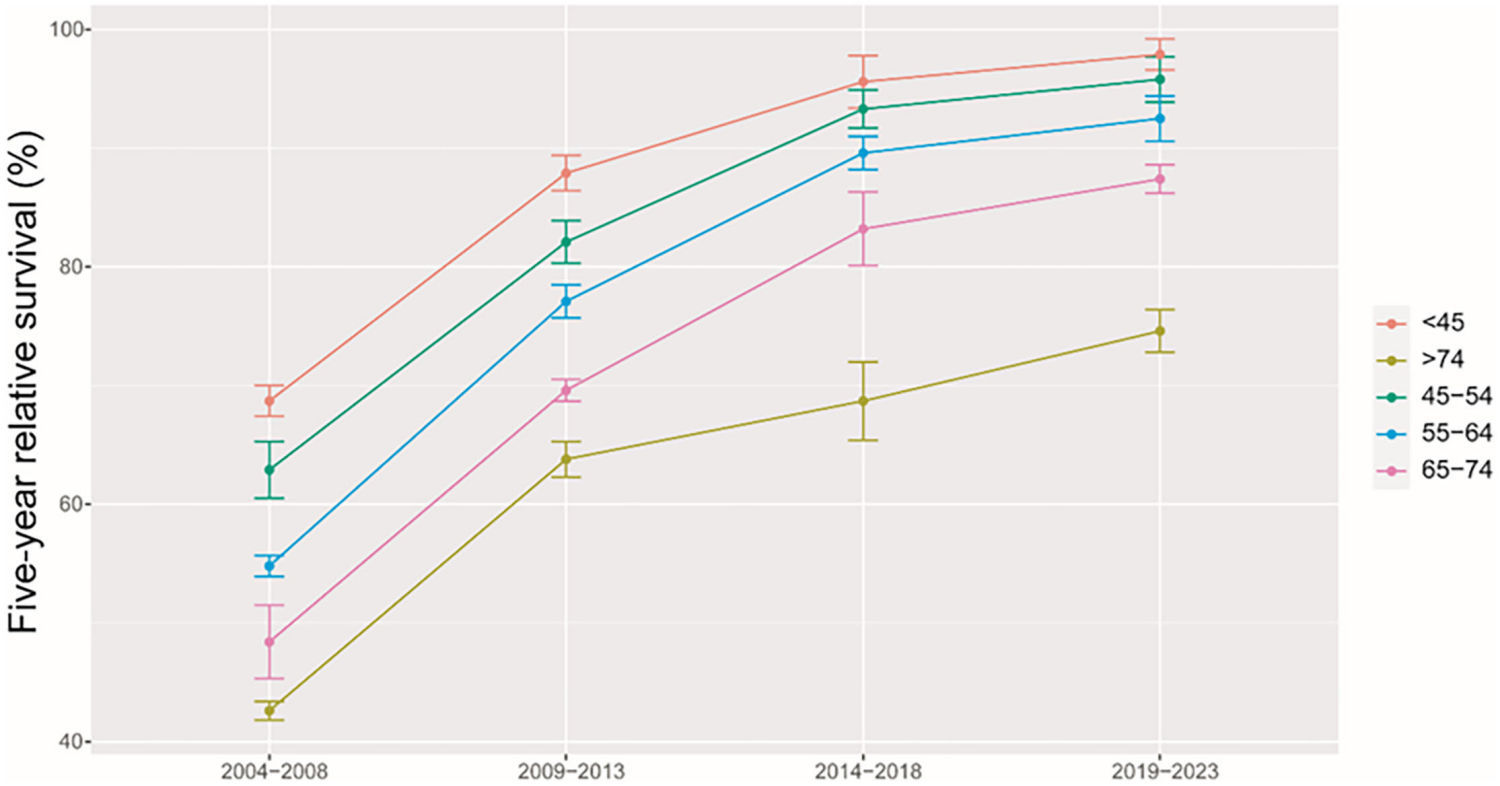
Trends of 5-year relative survival for patients with cervical cancer from Taizhou, eastern China during 2004–2008, 2009–2013, 2014–2018, and 2019–2023 by age at diagnosis.

We found a clear increasing trend of 5-year RS during 2004–2018 (2004–2008, 2009–2013, and 2014–2018) overall and on stratification by region (Figure 2) and age at diagnosis (Figure 3).

Using the data of 5-years RS during 2004–2018 and a model-based period analysis, we projected that the overall 5-year RS during 2019–2023 could reach 94.2% (Table 3). When stratified by age at diagnosis, we also found an age gradient, with 5-year RS decreasing to 97.9% for age <45 years and to 74.6% for age >74 years. Urban areas also had a higher 5-year RS compared to rural areas (94.8 vs. 90.2%).

**Table 3:** Projected 5-year relative survival of patients with cervical cancer during 2019–2023 in Taizhou, eastern China.

	**5-year relative survival (%)**
Overall	94.2
**Age at diagnosis (years)**
<45	97.9
45–54	95.8
55–64	92.5
65–74	87.4
>74	74.6
**Region**
Urban area	94.8
Rural area	90.2

## Discussion

We provided, for the first time in China, using period analysis, the most up-to-date (during 2014–2018) estimates of 5-year RS for patients with cervical cancer from Taizhou, eastern China, reaching 90.9%. When stratified by age at diagnosis, we found a clear age gradient for 5-year RS, declining to 95.6% for age <45 years and to 68.7% for age >74 years, while urban areas had higher 5-year RS compared to rural areas (92.9 vs. 88.6%). We found a clear increasing trend of 5-year RS during 2004–2018 overall and on stratification by region and age at diagnosis. Additionally, we estimate that the overall 5-year RS during 2019–2023 could reach 94.2%, using the data during 2004–2018 and a model-based period analysis.

We found that a 5-year RS during 2014–2018 for patients with cervical cancer from Taizhou, eastern China reached 90.9%. To our knowledge, this is the most up-to-date 5-year RS for Chinese patients with cervical cancer. Indeed, substantial improvements in survival for patients with cervical cancer could be likely attributed to improvements in surveillance, screening, and early detection. In our study, 58% of patients were diagnosed at <55 years (33% for 45–54 years), consisting of previous reports of peak incidence ranging 40–60 years old (12, 18). We found a clear age gradient for 5-year RS, declining to 95.6% for age <45 years and to 68.7% for age >74 years, which is in line with the previous report of a strong increase in mortality for those aged >75 years old (19).

We found that urban areas had higher 5-year RS compared to rural areas (92.9 vs. 88.6%). While the HPV vaccine was introduced into China in 2017, women in rural areas usually married at a young age and had an increased risk of HPV infection. Additionally, women in rural areas had limited access to screening programs for cervical cancer compared to women in urban areas. However, survival differences by region may narrow down shortly along with the implementation of China's rural cooperative medical system (20, 21).

We found a clear increasing trend of 5-year RS during 2004–2018 overall and on stratification by region and age at diagnosis. First, substantial investment in the health care system over decades by the Chinese government shall be the main reason (22). Additionally, increased access to effective treatment, medical care, and large-scale screening programs for cervical cancer at the population level may be other reasons.

Our study has several strengths and limitations. First, we provided, for the first time in China using period analysis, the most up-to-date (during 2014–2018) 5-year RS for patients with cervical cancer from Taizhou, eastern China. Second, we found that 5-year RS for patients with cervical cancer have improved greatly during 2004–2008, 2009–2013, and 2014–2018. Third, we projected the upcoming 5-year RS for patients with cervical cancer during 2019–2023. Although the projection is interesting, it was based on a pandemic-free scenario, and as we all noted, the pandemic may substantially limit the medical access both in urban and rural regions from 2020 until at least 2022 this year. Therefore, the projection may be influenced by the pandemic, providing less meaningful evidence in the real world. We also have limitations. First, we could not provide stratified survival data on stage, histology, and treatment of patients with cervical cancer. Nevertheless, population-based cancer registries commonly do not include clinical information on stage (such as TNM), histology, and treatment of patients with cancer. Hopefully, hospital-based cancer registries including detailed information on patients with cancer will be available soon in the future. Second, we only provided the most up-to-date survival data for patients with cervical cancer from Taizhou city. Therefore, further investigations using provincial or national data are also highly warranted.

## Conclusion

In this study, we provided, for the first time in China using period analysis, the most up-to-date 5-year RS for patients with cervical cancer from Taizhou, eastern China, reaching 90.9%. We found a clear increasing trend of 5-year RS during 2004–2018 overall and on stratification. Our finding of the projection during 2019–2023 reaches 94.2%, which may be influenced by the pandemic, providing less meaningful evidence in the real world, while the projection provided survival data to compare with estimated survival in the future. Our timely data on 5-year RS for patients with cervical cancer from Taizhou, eastern China is essential for the evaluation of early detection and screening programs for cervical cancer in Taizhou, eastern China. Further investigations using provincial or national data are also highly warranted.

## Data Availability Statement

The raw data supporting the conclusions of this article will be made available upon reasonable request to the corresponding author (TC).

## Author Contributions

TC was responsible for the study concept, design, and obtained funding. LW and TC acquired data. YC analyzed the data. HL, LL, LW, and TC drafted the manuscript. All authors revised it for important intellectual content. All authors contributed to the article and approved the submitted version.

## Funding

This work was supported by grants from the Ten-Thousand Talents Plan of Zhejiang Province (2021R52020), the Joint Key Program of Zhejiang Province-Ministry of Health (WKJ-ZJ-1714), and the Start-up Funds for Recruited Talents in Zhejiang Cancer Hospital. The funding agencies had no role in the design and conduct of the study; collection, management, analysis, and interpretation of the data; preparation, review, or approval of the manuscript; and decision to submit the manuscript for publication.

## Conflict of Interest

The authors declare that the research was conducted in the absence of any commercial or financial relationships that could be construed as a potential conflict of interest.

## Publisher's Note

All claims expressed in this article are solely those of the authors and do not necessarily represent those of their affiliated organizations, or those of the publisher, the editors and the reviewers. Any product that may be evaluated in this article, or claim that may be made by its manufacturer, is not guaranteed or endorsed by the publisher.

## Abbreviations

DCO: proportion of death certificate only
RS: relative survival
ICD-10: International Classification of Diseases 10th Revision
GLM: the generalized linear model.
