# Supplementary material for: Timely Estimates of 5-Year Relative Survival for Patients With Cervical Cancer: A Period Analysis Using Cancer Registry Data From Taizhou, Eastern China
Source: Front Public Health. 2022 Jul 25;10:926058. doi: 10.3389/fpubh.2022.926058 (PMC9358018; doi:10.3389/fpubh.2022.926058)
Supplement: Supplementary file 1 [file Data_Sheet_1.docx]

**Supplementary**

**Period analysis was used to calculate 5-year RS**

In a first step, we assessed 5-year RS of patients diagnosed in 2014–2018 by period analysis and divided patients into the newly diagnosed patients during 2014-2018 and the patients diagnosed from 2009 to 2013 but still alive within 2014-2018. The period analysis uses survival experience observed in a specified calendar period, which included left censoring of observations and survival observations are right truncated at the end of the calendar period. Additionally, the method calculated the 1-year RS *Si* at the *i* year of follow-up based on the collection of a life table from cancer registries. The formula can be written as:

$$\boldsymbol{S}_{\boldsymbol{i}}\mathbf{=}\mathbf{1}\mathbf{-}\frac{\boldsymbol{d}_{\boldsymbol{i}}}{\boldsymbol{n}_{\boldsymbol{i}}\mathbf{-}{\boldsymbol{c}_{\boldsymbol{i}}}/\mathbf{2}}$$

In this formula, where *n_i_* denoted the population at the beginning of the *i* year of follow-up, *d_i_* denoted the number of deaths at the end of *i* year of follow-up, and *c_i_* denoted the number of censored data in *i* year. The estimate of survival by the end of follow-up year *k* (*s_k_*) was derived from multiplying the one-year survival rate of the conditions of *k* years. The formula can be written as:

$$\bar{\boldsymbol{S}_{\boldsymbol{k}}}\mathbf{=}\prod_{\boldsymbol{i}\mathbf{=}\mathbf{1}}^{\boldsymbol{k}} \boldsymbol{S}_{\boldsymbol{i}}$$

RS was the ratio of the observed survival divided by the expected survival. The formula was given as:

$$\boldsymbol{R}_{\mathbf{i}}\mathbf{=}\frac{\bar{\boldsymbol{S}_{\boldsymbol{k}}}}{\boldsymbol{S}_{\boldsymbol{k}}^{\mathbf{*}}}$$

Where $\bar{S_{k}}$ denoted observed survival, and$S_{k}^{*}$ denoted the expected survival, which is calculated using the Ederer II method. When *k*=5, the estimates of 5-year RS derived from this formula. According to the Greenwood method, the point estimate of the RS and its standard error were calculated.

Next, the model-based period analysis was used to predict the 5-year RS of patients in the four counties of Taizhou City, Zhejiang Province between 2019 and 2023, with further analysis stratified by sex, age at diagnosis, and region. A generalized linear model (GLM) based on period analysis and complete cancer registries was established to estimate the survival of patients, analyze trends in survival, and predict future survival. The method was based on the premise that survival changes uniformly over time. Theoretically, the accuracy of survival analysis increases with longer time spans when survival rates remain constant or increase uniformly; on the contrary, when the survival rate rises unevenly or even decreases, extend the time span and reduce the accuracy of survival analysis. The model-based period analysis included the data from 2004-2008,2009-2013, and 2014-2018, respectively. The conditional 1-year survival was calculated first, and then a regression model (Poisson regression or binomial regression) with the follow-up year as the independent variable and 1-year survival as the dependent variable was established [11]. The estimates of conditional relative survival for each combination of follow-up year *i* and calendar year *j* was as follow:

$$\boldsymbol{r}_{\boldsymbol{ij}}\mathbf{=}\boldsymbol{exp}\left( \mathbf{-}\boldsymbol{exp}\left( \boldsymbol{\alpha}_{\boldsymbol{i}}\mathbf{+}\boldsymbol{j}\boldsymbol{\times}\boldsymbol{\beta} \right) \right)$$

In this formula, *j* represented the calendar years, which were coded in such a way that *j* = 0 for the first calendar year and *j* = k for the k+1 calendar year of the calendar period included in the modeling. *i* represented the follow-up-year, for example, within 2004-2008, where 2004 corresponded to *i* =1 and 2005 corresponded to *i* =2, and so on.

Moreover, an estimate of cumulative 5-year relative survival for each calendar year *j* was given:

$$\boldsymbol{R}_{\boldsymbol{j}}\mathbf{=}\prod_{\boldsymbol{i}\mathbf{=}\mathbf{1}}^{\mathbf{5}} \boldsymbol{r}_{\boldsymbol{ij}}\mathbf{=}\prod_{\boldsymbol{i}\mathbf{=}\mathbf{1}}^{\mathbf{5}} \boldsymbol{exp}\left( \mathbf{-}\boldsymbol{exp}\mathbf{}\left( \boldsymbol{\alpha}_{\boldsymbol{i}}\mathbf{+}\boldsymbol{j}\boldsymbol{\times}\boldsymbol{\beta} \right) \right)$$
